# The Effects of Hispanic-Focused Dementia Education on Nursing Students’ Awareness, Knowledge, and Confidence

**DOI:** 10.1093/geroni/igaf122.2927

**Published:** 2025-12-31

**Authors:** Jessica Sanchez, Aleatha Rossler

**Affiliations:** Texas Woman’s University, Dallas, Texas, United States; Texas Woman’s University, Dallas, Texas, United States

## Abstract

**Background:**

Hispanic Americans are 1.5 times more likely to develop dementia than non-Hispanic White Americans, yet experience a greater delay in diagnosis. The cause for delay is multifactorial, including discrimination when seeking healthcare, predisposition to diseases that increase dementia risk, language and financial barriers, and lack of knowledge. Nurses can play a critical role in addressing these barriers, yet nursing curricula lack formal programs to prepare students for this topic.

**Purpose:**

To evaluate the effects of a Hispanic-focused dementia educational intervention on nursing students’ awareness, knowledge, and confidence in caring for this patient population.

**Methodology:**

A single-group, pretest-posttest design was employed, utilizing a convenience sample of undergraduate nursing students. Participants completed assessments to measure their awareness, knowledge, and confidence levels before and after the educational intervention.

**Results:**

There was a statistically significant increase in awareness of prevalence of health conditions that increase dementia risk (p = 0.002) and prevalence of dementia (p = 0.02) within communities of color; increase in confidence in ability to educate Hispanic patients diagnosed with dementia (p = 0.003); and increase in the mean knowledge of dementia within the Hispanic population.

**Conclusions:**

These findings support the data-driven change of integrating this educational intervention into the nursing curriculum to prepare nurses for delivering culturally competent care. This initiative is an important step to empower future nurses to improve health outcomes and ensure equitable care for vulnerable populations in professional practice.

